# Social media in knowledge translation and education for physicians and trainees: a scoping review

**DOI:** 10.1007/s40037-019-00542-7

**Published:** 2019-12-13

**Authors:** Teresa M. Chan, Kristina Dzara, Sara Paradise Dimeo, Anuja Bhalerao, Lauren A. Maggio

**Affiliations:** 1 McMaster University, Hamilton, Ontario Canada; 2 Harvard Medical School and Massachusetts General Hospital, Boston, USA; 3 Prisma Health-Upstate Department of Emergency Medicine, University of South Carolina, Greenville, South Carolina, USA; 4 University of Toronto, Toronto, Canada; 5 Uniformed Services University of the Health Sciences, Bethesda, MD USA

**Keywords:** Social media, Knowledge translation, Medical education

## Abstract

**Introduction:**

The use of social media is rapidly changing how educational content is delivered and knowledge is translated for physicians and trainees. This scoping review aims to aggregate and report trends on how health professions educators harness the power of social media to engage physicians for the purposes of knowledge translation and education.

**Methods:**

A scoping review was conducted by searching four databases (PubMed, Scopus, Embase, and ERIC) for publications emerging between 1990 to March 2018. Articles about social media usage for teaching physicians or their trainees for the purposes of knowledge translation or education were included. Relevant themes and trends were extracted and mapped for visualization and reporting, primarily using the Cook, Bordage, and Schmidt framework for types of educational studies (Description, Justification, and Clarification).

**Results:**

There has been a steady increase in knowledge translation and education-related social media literature amongst physicians and their trainees since 1996. Prominent platforms include Twitter (*n* = 157), blogs (*n* = 104), Facebook (*n* = 103), and podcasts (*n* = 72). Dominant types of scholarship tended to be descriptive studies and innovation reports. Themes related to practice improvement, descriptions of the types of technology, and evidence-based practice were prominently featured.

**Conclusions:**

Social media is ubiquitously used for knowledge translation and education targeting physicians and physician trainees. Some best practices have emerged despite the transient nature of various social media platforms. Researchers and educators may engage with physicians and their trainees using these platforms to increase uptake of new knowledge and affect change in the clinical environment.

**Electronic supplementary material:**

The online version of this article (10.1007/s40037-019-00542-7) contains supplementary material, which is available to authorized users.

## What this paper adds

This paper comprehensively maps the evolving landscape of how social media is changing the way medical educators use social media to translate knowledge or educate end-user physicians and their trainees. The authors highlight gaps and opportunities for further scholarship and research, while succinctly describing the current state of the literature.

## Introduction

For over a decade, social media has become an increasingly powerful tool harnessed by physicians to disseminate knowledge to one another, their learners, patients and the public [1–3]. Defined broadly, social media includes technology-mediated platforms that facilitate individual users to create and distribute content (both user-generated and user-curated) to virtual communities of practice [4, 5]. These platforms can range from collaborative authorship platforms such as wikis (e.g., Wikipedia) to single-author dialogue platforms such as micro-blogging (e.g., Twitter) [6–13].

Many potential uses of social media have emerged, and increasingly journals, scientists, and researchers are using it for reaching their end users and engaging in education and knowledge translation. Knowledge translation is defined as the communication between scientists, healthcare professionals, educators, and journals to convey information [2]. As personal use of social media has grown, so has its use for purposes of knowledge translation. Initially, this included mostly dissemination via blogs, wikis, and podcasts, but more recently has evolved to include other media, such as microblogging (e.g., Twitter), social networks (e.g., Facebook, LinkedIn), and video-based outlets (e.g., YouTube). Increasingly, academic journals are leveraging social media to encourage readers to engage with their materials [14] and are experimenting with using online social media conversations [15, 16], infographics [17, 18] and podcasts [17, 19, 20] to increase readers’ awareness of new publications. In many ways, knowledge translation is a scientist-based description of a phenomenon that overlaps with the individual end-user’s education, especially when they intersect within social media platforms. As such, for the purpose of our scoping review we have coalesced the concepts of education and knowledge translation, since we felt that we would be unlikely to make true distinctions in our synthesis of the literature.

While there have been some reviews about the use of social media for education [21–24], none have sought to fully encompass the breadth of how these technologies have affected the full spectrum of education, including the continuing professional development of practising physicians. Interestingly, in both postgraduate and continuing medical education, the lines between knowledge translation and education often blur since new knowledge that is being disseminated or translated by scientists is often being consumed by trainees and practising physicians as part of their continuing lifelong learning.

The purpose of this scoping review is to describe the breadth of social media-based technologies used for knowledge translation, as well as dissemination strategies used by scientists, educators, and journals to educate clinical colleagues. We aim to map the landscape of knowledge translation and education via social media in academic medicine. Through this scoping review, we hope that by examining and synthesizing the work that has come so far, we can identify trends and best practices for engaging in knowledge translation and education in today’s social media-laden world.

## Methods

We conducted a scoping review guided by Arksey and O’Malley’s framework, which features six steps as outlined below [25]. A scoping review methodology was selected to enable us to characterize the current literature and identify gaps. Our initial database extraction occurred on 27 March 2018; the remaining phases of our study were conducted over the following year.

### Step 1: Identifying the research question

In this review, we focused on three key questions: 1) What are the most frequently reported knowledge translation and education strategies using social media? 2) How are these strategies most frequently reported? 3) Has the effectiveness of these social media strategies been reported, and if so, what were the primary outcomes?

Our questions were generated through an iterative process of discussion within our research team and shaped by consultation of the literature. The research team also drew upon its extensive experience with various social media platforms and conducting research in this domain.

### Step 2: Identifying relevant studies

Based on our research questions, LAM, a health professions education researcher and information scientist, designed a PubMed search using Boolean operators to combine medical subject heading terms and keywords. Search terms included, but were not limited to: social media, Facebook, Twitter, Web 2.0, medical education, physicians, medical students (See Supplement 1 of the online Electronic Supplementary Material for the complete search strategies).

An independent medical librarian peer reviewed the PubMed search strategy to ensure its comprehensiveness. After discussion with LAM, the medical librarian translated the PubMed search for optimal use in Scopus, Embase, and ERIC, and ran the searches in all databases on 27 March 2018. No limits related to language or date range were applied. Upon retrieval, results from all databases were deduplicated and exported for management into Excel.

### Step 3: Study selection

The author team participated in multiple conference calls to determine inclusion criteria. During these calls, we iteratively reviewed articles discussing relevance to the research questions until group consensus was reached. Ultimately, we decided to focus on articles that included social media as a communication tool targeted towards physicians and/or medical trainees. We excluded publications reporting on physician-patient communications or those purely focused on online professionalism, personal reflection without a practice improvement focus, and physician/trainee identity formation. Lastly, we excluded publications simply describing social media as a broadcasting tool.

To facilitate study selection, we restricted database searches to journal articles and conference proceedings, including white papers, indexed in our selected databases. We excluded scholarly blogs or websites because they are not uniformly indexed across the various databases. Citations, selected for full-text review, not in English were translated by the lead author (TC) using Google Translate.

### Step 4: Charting the data

In alignment with our research question and informed by the Best Evidence in Medical Education data extraction tool [26], we iteratively designed a data extraction sheet in Excel. We based portions of the extraction tool on the Cook, Bordage, and Schmidt framework for types of scholarship (description, justification, and clarification) [27]. Additionally, we classified outcomes measured in empirical studies according to Kirkpatrick’s evaluation framework [28, 29]. All authors piloted this extraction sheet using a randomly selected sample of 10 articles. Upon finalizing the extraction tool, it was operationalized in Google Forms (Mountainview, CA) for ease of data entry. (For the extraction tool see Supplemental Appendix 1).

### Step 5: Collating, summarizing and reporting the data

The list of studies for extraction was divided equally amongst the authors. Each author was responsible for including or excluding the study as well as extracting relevant information from included studies. Studies that were classified as ‘maybe include’ were reviewed by a second author for a final decision. Studies not in English were also included and underwent the same review process. Google Translate (Mountainview, CA, USA) was used to translate the content and decide accordingly.

Throughout our extraction process, we identified key themes related to the educational use of social media for knowledge translation. Initially, based on our familiarity with the field and literature, we started with a working list of potential key concepts that sensitized our approach. However, we also iteratively added to this list throughout data extraction. The themes and the analysis of our extraction were reviewed by our consulting experts to ensure the rigour of the findings (see below).

### Step 6: Consultation

After our data extraction, we consulted three researchers who have published at least five publications on social media use in relation to knowledge translation and education and act as active practitioners (e.g., heading an online education blog, serving as social media editor for a major medical journal). These consulting researchers were known to the lead investigator (TMC) and had co-authored multiple papers in the area of social media-based knowledge translation and education.

Each consultation consisted of one-on-one interviews, with TMC walking them through our key findings (e.g., she presented our collated and summarized data regarding the literature that we found). The themes, tables, and findings were discussed with these individuals, and their feedback on our findings was sought. Specifically, we asked each individual if our findings were consistent with their own understanding of the status of the literature and if there were any specific gaps in our analyses. Further analyses suggested by the experts were incorporated into our final manuscript, specifically in the results in one of our tables (specifically indicated in the results section) and discussion sections.

## Results

**Fig. 1:**
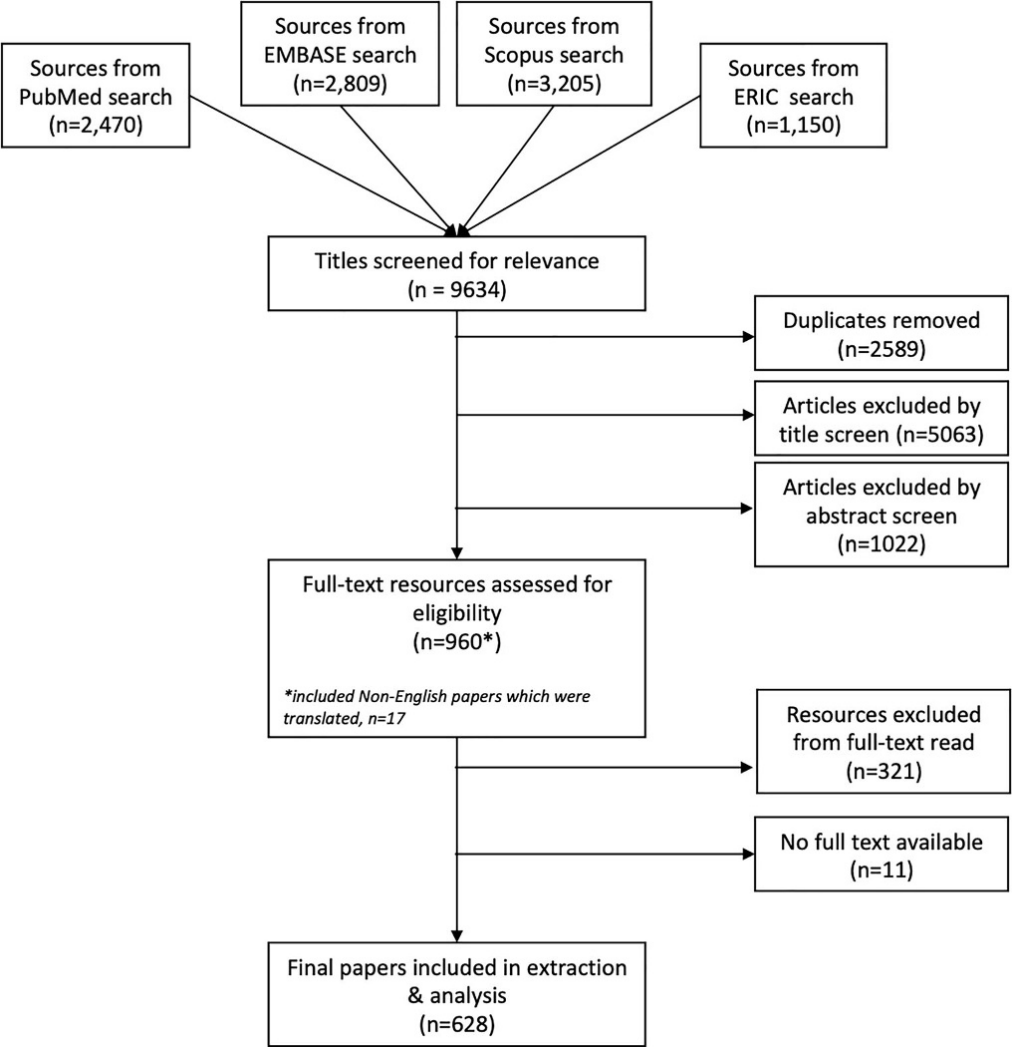
Flow diagram of search and inclusion process

Our search retrieved 9634 potential citations, which based on our criteria were reduced to 628, the full-texts of which were analysed (See Fig. 1 for a flow diagram of the results). We were unable to locate the full-text of 11 publications despite searching 5 separate institutional libraries and emailing corresponding authors three times. These publications were excluded.

Many of the publications (*n* = 228) were not geographically anchored (e.g., they did not refer to a specific region of the world). For the remainder, the majority were in North American (*n* = 187) or European (*n* = 88) contexts, although there were publications from many other jurisdictions [Asia (*n* = 31), Australia/New Zealand (*n* = 20), Middle East (*n* = 11), Africa (*n* = 5), South America (*n* = 4)].

### Prevalence over time

**Fig. 2:**
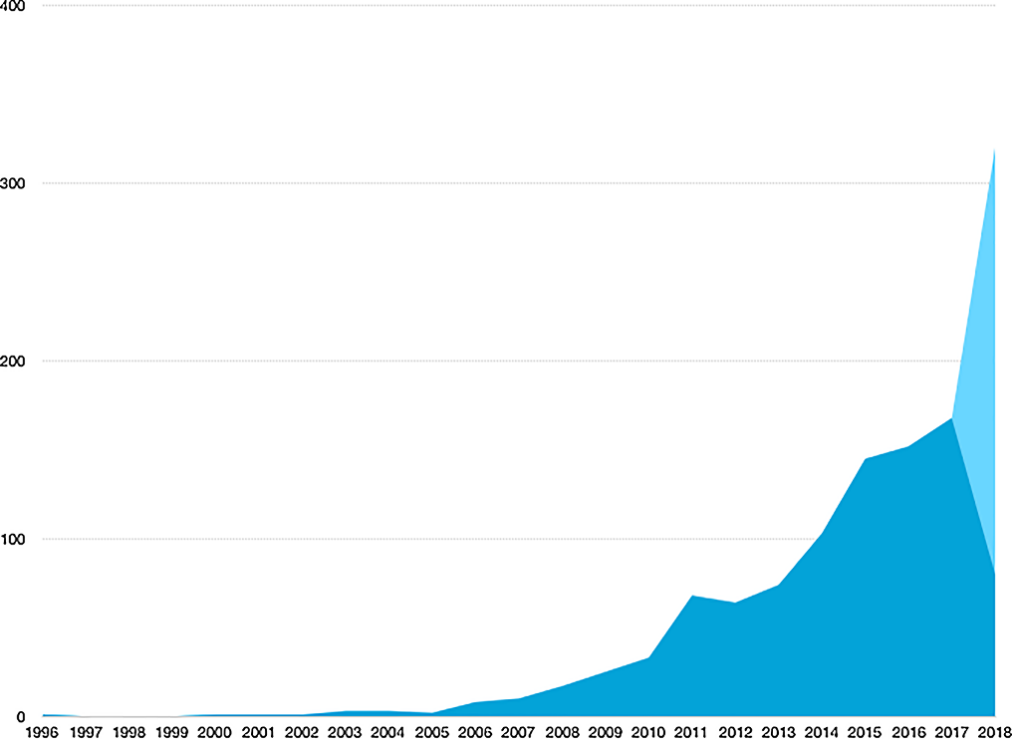
Number of publications on social media knowledge translation and education for physicians and medical trainees. *NB* The 2018 data is extrapolated from projected data since our search included only the first 3 months of 2018. To generate the year-end projection, the number of papers published in the first 3 months was multipled by four to yield the anticipated total number of papers by year’s end

Over the past decade, publications featuring social media for knowledge translation and education of physicians and medical trainees have increased (See Fig. 2).

### Specialties of origin

We identified a range of specialties, with surgical specialties most often represented. (See Table 1 for specialties or groups of specialties that were included in more than 10 publications.)

**Table 1:** Medical specialties or groups of specialties that were included in more than ten publications

Specialty or grouping	# of Articles
Surgery (including specialties and subspecialties) Urology (*n* = 24) Plastics/Burns/Aesthetics (*n* = 18) Orthopaedics (*n* = 16) Neurosurgery (*n* = 10)	138
Medicine (including hospitalist, internal medicine) and subspecialties Nephrology (*n* = 10)	85
Emergency medicine (including paediatric emergency medicine (*n* = 3))	59
Radiology (including subspecialties)	24
Dermatology	18
Primary care (including family medicine, general practice, palliative care, travel medicine)	18
Anaesthesia (including pain medicine, and critical care anaesthesia)	16
Pathology (including subspecialties)	13
Psychiatry	10

### Target audiences

The majority of articles targeted attending (fully qualified and practising) physicians (*n* = 346) and postgraduate trainees (e.g. residents and fellows, *n* = 242). Of the articles, 195 described interventions aimed to educate or disseminate new knowledge to medical students in addition to other learners, with 20 of these publications focusing on preclinical basic science, and 11 focusing exclusively on undergraduate medical education. Eighty-three articles addressed our target audiences plus at least one other health professional group (e.g., nurses, dentists). The majority of papers within all of the groups tended to address similar issues (i.e., using social media for learning, uptake and usage), although the articles targeting trainees/students tended to have more content regarding professionalism (many of which were excluded in our final analysis).

### Social media strategies used for knowledge translation and education

#### Prevalence of types of social platforms

A wide range of social media platforms were featured, including Twitter (*n* = 157), blogs (*n* = 104), Facebook (*n* = 103), podcasts (*n* = 72), video archival platforms (e.g. YouTube, *n* = 68), and Wikipedia (*n* = 57) (See Table 2 for a comprehensive platform listing). The majority of articles focused on the technology itself (77.5%, *n* = 487) (e.g., an article that introduces a medical specialty to Twitter [30, 31]), whereas the remainder focused on the individuals using the technology (22.5%, *n* = 141), such as the rate at which residents used podcasts or blogs [10, 32].

**Table 2:** Types of social media represented

Platform (Number of Articles)
*Open social media platforms (# of articles)*
Twitter (157)
Facebook (103)
Wiki (57)
LinkedIn (25)
Google+ (15)
Instagram (10)
Flickr (9)
ResearchGate (3)
Reddit (3)
mySpace (2)
Pinterest (2)
Snapchat (1)
*Multimedia with ability to interact (# of articles)* *(e.g., messaging, commenting, correspondence)*
Blogs (104)
Podcasts (72)
Video Archival (68) (e.g., YouTube, Vimeo, etc.)
RSS Feeds (4)
iTunesU (1)
Scribd (1)
*Direct peer-to-peer contact (# of articles)*
WhatsApp (19)
Video Chat (14)
WeChat (7)
Texting (2)
*Other closed platforms (# of articles)*
Specific closed networks (24) (e.g., Doximity, Moodle, Slack)
Google docs/Collaborative document sharing (7)
Social Bookmarking (7)
Digital Textbook (4)
SlideShare (2)

#### Strategies described using social media platforms to engage in knowledge translation and education

Reviewed publications described many social media strategies. These strategies focused on ways to push out information or foster engagement. For example, several studies described organized efforts by journals, professional societies, and individuals to push journal articles to their audiences, such as creating a podcast to showcase a recent article. Other common strategies focused on fostering interaction between individuals. For example, multiple residencies reported using social media to facilitate engagement around journal articles using a virtual journal club format. In several cases, strategies contained both efforts to push information and foster engagement. Table 3 features examples of identified strategies. This table specifically arose because of our expert consultation process.

**Table 3:** Social media strategies for knowledge translation and education

Push strategies with occasional social media engagement opportunities	Engagement strategies with a focus on fostering interaction between individuals	Blended strategies using ‘push’ and engagement
– Blog for dissemmination, communication, and education – Distributing Visual Media (e.g. Visual Abstract, Infographics, Instagram accounts) – Podcasts – Webcasting (e.g. YouTube Channel) – Dedicated Organizational Social Media Accounts (e.g., a journal’s Twitter account, a residency program’s instagram account)	Social media discussions – Online Case-based or – Problem-based learning (connectivist Massive Open Online Course) – Twitter Journal Clubs & other Tweet Chats – Facebook groups/pages – WhatsApp-based teaching/discussions – Mobile quizzing (e.g. board exam review, image review) – Wiki-based collaborations – Cultivating online/virtual Communities of Practice – Hashtag creation (e.g. #FOAMed, #WomenInMedicine, #PlasticSurgery)	– Live-tweeting at conferences – Virtual networking and mentorship – Formal publications based on social media discussions (e.g. Online journal club proceedings)

#### Types of social media scholarship in knowledge translation and education

The bulk of included publications (*n* = 242) were descriptive studies of physicians’ social media usage for the purposes of knowledge translation and education. Another prominent scholarship type (*n* = 192) was conceptual or narrative reviews describing the application of social media for connecting physicians and their trainees. Innovation reports were the next most prevalent type of scholarship (*n* = 122). A minority of studies included elements of clarifying between types/formats of social media for knowledge translation and education (*n* = 5), critical appraisal of social media-based content (*n* = 5), and integrative reviews of social media (*n* = 3). Table 4 lists types of scholarship in knowledge translation and education.

**Table 4:** Types of scholarship in the social media for knowledge translation and education literature

Type of scholarship (*n*)	Explanation of scholarship type
Descriptive study (*n* = 242)	Description of a population and their habits. In our database, usually describing the usage patterns of technology (e.g. how many physicians various social media platforms)
Conceptual piece (*n* = 192)	Narrative review, opinion, editorial, commentary, or letter to the editor advancing theory
Innovation report (*n* = 122)	Description of one specific intervention at the local level or initial trial, richly described with satisfaction or reaction data
Justification study (*n* = 45)	Compares one educational intervention with another to assess whether the intervention works compared with a known standard
Online journal club proceedings (*n* = 19)	Online discussions were facilitated via social media, and proceedings were reported in a formal peer reviewed publication
Clarification study (*n* = 5)	Either testing theory-based predictions (e.g. experimental quantitative approaches) or generating new theory (e.g. mixed or qualitative approaches) and building upon prior research
Critical appraisal of online materials (*n* = 5)	Systematically rating the quality of websites, blogs, podcasts or other social media materials
Systematic or other integrative reviews (*n* = 3)	This category includes systematic reviews, meta-analyses, scoping reviews, and other structured integrative work, but excludes non-structured review (e.g. narrative reviews or commentaries). Scientific review summarizing the results of available evidence on a topic

#### Empirical studies

The majority of empirical studies (*n* = 353) applied quantitative methods (79.9%, *n* = 282). Forty-four studies utilized mixed methods (12.5%, *n* = 44) and a minority featured qualitative methods (7.5%, *n* = 27). Five studies critically appraised online content and two reported consensus building using modified Delphi methods.

Methods used in studies about knowledge translation and education for physician audiences were varied. The predominant study design was the quantitative survey (*n* = 180 studies), which was often mixed with other methods such as free-text survey questions. Usage analytics were also popular (*n* = 79). Other common study types included applying objective observations or text analysis of substantive written texts (e.g., blog posts or archived narratives), interviews, and micro-text (e.g. Tweet) analysis. Table 5 reports all methods used.

Within empirical studies, multiple themes were discerned via our analysis of these types of articles. We identified the feasibility of social media for practice improvement as the most prevalent theme (*n* = 330). Multiple publications also described specific elements of a particular technology (*n* = 327), such as podcasts or e‑learning. Examples of these studies were: a) Lien and colleagues comparing blog posts to podcasts; both groups showed increased knowledge retention, with no preference for one media over the other [33]; b) multiple studies examined the use of mixed media (podcasts, video) for medical students and resident translation of learning, ultimately concluding that learners rated the media tool more highly than traditional learning [34, 35]; c) other studies have examined use of Twitter as a study aid or engagement tool in student courses, but have not shown improved performance [36, 37].

Table 6 features major themes identified and provides a reference to exemplar papers. Some of the rarer themes included: challenges and pitfalls aside from professionalism (*n* = 5), humanism (including medical humanities and reflective practice, *n* = 3), costs of the innovation (*n* = 2), correlation of social media with bibliometrics (*n* = 2), health policy change and advocacy (*n* = 2), informatics (*n* = 1), and mentorship (*n* = 1).

**Table 5:** An overview of the methods used study social media knowledge translation and education

Types of Methods Used	Number of Publications
Surveys (quantitative—number-based, questions Likert scales, other scales)	180
Usage analytics (Web or Social Media Platform analytics—Pageviews, Number of Tweets, etc.)	79
Objective observations/Tests	47
Surveys (qualitative—open ended questions)	40
Substantive written texts (narratives, reflections, blog posts)	34
Interviews	20
Micro-text analysis (e.g. Tweet analysis)	18
Anecdotes and exemplar case studies	16
Focus Groups	10
Social media profile review	9
Critical appraisal of online content	8
Ethnographic approaches (e.g. Observations with field notes, online observation)	4
Delphi study	3
N/A (i.e. was a commentary or narrative review)	192

The total in this table is greater than the total number of publications from which we extracted since some studies included multiple types of methods (e.g., studies might incorporate both quantitative and qualitative survey elements)

**Table 6:** Major themes in the social media for knowledge translation and education literature

Themes (# of articles)	Exemplar article
Practice Improvement (*n* = 330)	Alam F, et al. E‑learning optimization: The relative and combined effects of mental practice and modeling on enhanced podcast-based learning—a randomized controlled trial. Adv Health Sci Educ. 2016;21:789–802 [38]
Description of technology (*n* = 327)	Sugawara Y, et al. Medical Institutions and Twitter: A Novel Tool for Public Communication in Japan. JMIR Public Health Surv. 2016;2(1) [39]
Community of practice (*n* = 61)	Stewart S, Abidi S. Applying Social Network Analysis to Understand the Knowledge Sharing Behaviour of Practitioners in a Clinical Online Discussion Forum. JMIR. 2012;14(6) [40]
Critical appraisal of the online content (*n* = 53)	Wolbrink T, et al. The Top Ten Websites in Critical Care Medicine Education Today. J Int Care Med. 2018;34:3–16 [41]
Professionalism addressed in addition to other themes (*n* = 48)	Chretien K, et al. Physicians on twitter. JAMA. 2011;305:566–8 [42]
Informing evidence-based practice (*n* = 43)	Joshi N, et al. Social Media Responses to the Annals of Emergency Medicine Residents Perspective Article on Multiple Mini-Interviews. Ann Emerg Med. 2014;64:320–5 [43]
Usage patterns & demographics (*n* = 21)	Chan TM, et al. Creating, curating, and sharing online faculty development resources: the medical education in cases series experience. Acad Med. 2015;90:785–9 [44]

### Outcomes measured in social media for knowledge translation and education studies

Most (134; 70.1%) articles measuring outcomes were at the acceptability level. For example, in one study researchers developed audiovisual podcasts, which were reviewed by medical students to assess their usability [45]. Forty-eight studies (24.7%) measured knowledge acquisition. For example, one research team conducted a prospective, nonblinded, three-arm randomized trial to compare use of Wikipedia, UpToDate, and a digital textbook for knowledge acquisition among pre-clerkship students to assess knowledge acquisition [46]. Six studies (3.1%) measured behavioural change. Finally, four studies (2.0%) measured organizational or patient outcomes. For example, one study compared the use of emergency department communications and peer-to-peer learning via WhatsApp and standard telephone for educating peers during consultations [47].

### Diffusion of an innovation through scholarship

**Fig. 3:**
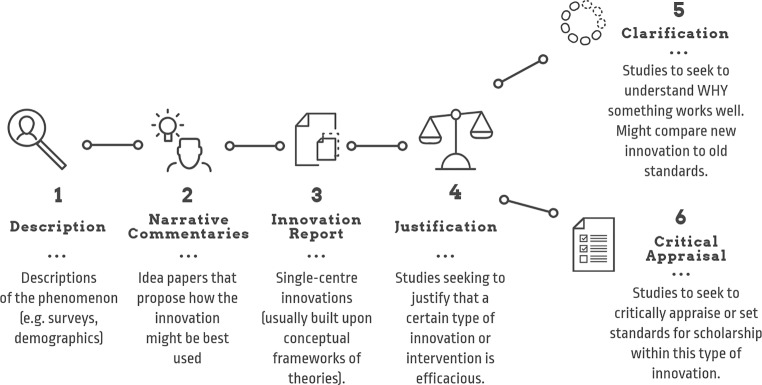
Diffusion of innovation through scholarship

There appeared to be a diffusion of innovation that has moved into more scholarly avenues—a *diffusion of innovation through scholarship*. Akin to Rogers’ framework for diffusions of innovation [48, 49], we found a pattern in our review of the literature. We have assembled our findings into a new conceptual framework, which highlights specifically how educational innovations seem to diffuse through the scholarly system. For instance, we observed the trend of narrative papers commenting about the potential and usage of social media, moving into more descriptive papers about the phenomenon via surveys and demographics-based studies, with patterns of usage and preferences for usage appearing in tandem with the more narrative literature. Soon after, the one-off, single-centre innovation publications emerged—variably describing interventions, reporting new ideas for application, with a mind for others to replicate and reproduce. After the appearance of descriptive publications come the justification articles (which seek to substantiate why a certain innovation is worthy) [27]. Finally, there are the clarification papers (which seek to clarify ‘what works’) [27] and the critical appraisal publications, which seek to set standards and examine the innovation via a more critical lens. Fig. 3 depicts the scholarly sequence that social media for knowledge translation and education seemed to have appeared in our review; the stages were not discrete and often overlapped as the field tended to mature over time.

## Discussion

In the past few decades, there has been a veritable boom of scholarly work describing the ways social media can be used for knowledge translation and education; our scoping review maps the trends of these uses of social media. In these next sections, we discuss the social media landscape highlighting trends and possible future research directions.

The landscape of social media for knowledge translation and education has shifted from simple descriptions to more nuanced clarifications around best practices and bringing an element of criticality to its implementation. More recent publications seek to justify and clarify the use of social media for knowledge translation and education tended to aim for higher Kirkpatrick outcomes (e.g., Level 4—measuring organizational changes, Level 3—behavioural changes, Level 2—knowledge change) rather than simply reporting acceptability (Kirkpatrick Level 1) [29]. Likely, it is time for scholars to shift from simply engaging in descriptive or conceptual papers focused on highlighting specific social media platforms towards more justification and clarification work—after all, our search shows that platforms tend to rise and fall (e.g., MySpace is barely mentioned, while the dominance of Twitter or Facebook may someday similarly decline).

### The emerging power of online communities

Sixty-one publications addressed online communities. The growth of the *Free Open Access Medical education* (FOAM, or #FOAMed) movement has fostered the growth of hundreds of English-language blogs and podcasts in two specialties alone (emergency medicine and critical care) [1]. Fuelled by the growing FOAM movement [1, 50], they have started to embrace alternative outlets for knowledge translation and dissemination: for example, exchanging information and ideas about new research, evidence, or guidelines by tweeting and using specialty specific hashtags. Whether to share conference pearls [51–54], discuss best practices via a podcast [55], or utilization of online platforms for faculty teacher development [44, 56], there is now an unprecedented ability to share information beyond traditional venues (e.g. conferences or publications).

Enabled by networks such as those that have sprung up around FOAM, virtual communities of practice can enable members to more quickly share common knowledge [57, 58]. There are growing numbers of online communities of practice, which not only allow, but encourage educators to increase peer visibility and recognition. They can also be harnessed to amplify messages, allowing for a faster dissemination of work, theoretically leading to increased immediate impact [57]. Because of the growth of virtual communities of practice [4, 5, 59, 60] which facilitate innovative knowledge translation, new educational approaches, and dissemination practices, the best practices for using social media to translate new knowledge to other healthcare professionals is a moving target.

## Limitations

Our study has several limitations. First, although we searched several databases, attempting to optimize our search strategies for comprehensiveness, it is possible we inadvertently missed citations. Secondly, we did not assess publication quality or impact. Instead, we aimed to include rather than exclude publications to provide a comprehensive overview to inform future scholarship. We also used Google Translate to translate non-English papers. Had we conducted a more complex textual review, we might have utilized another tool or translation services, which more effectively captures the nuance of language. However, for the purposes of our data extraction (Appendix 1), we only extracted surface features so Google Translate was appropriate for our purpose.

Purposefully, we broadly defined social media to avoid preconceived notions of what is traditionally deemed as ‘social media’ per popular definitions. We aimed to include technology that linked two or more individuals in direct exchanges and engagement, excluding one-way communication methods (pure broadcasting via e‑modules) or more traditional formats of correspondence such as e‑mail and listservs. Additionally, we excluded social media use as it relates to professionalism, which was a popular topic in the literature but did not embody our primary study purpose. Also, as noted in the introduction there is considerable overlap between knowledge transfer and education. This finding was underscored by how these two concepts were intertwined in the included studies; it was impossible to disentangle these two concepts, so we addressed them together. Future researchers might consider focusing on the different ways in which medical education addresses these two concepts in relation to social media. Finally, our review was limited to social media which targeted physicians and trainees, and as such cannot comment on other groups of healthcare providers or scientists.

## Next steps: Gaps & opportunities

In our review, we observed prevalent themes and those that have been less explored, but are ripe for future research. We identified that only two publications examined costs related to social media in the context of knowledge transfer and education, which could be considered a major challenge for this field [61, 62]. While most social media platforms are free to use (e.g., there is no charge operating a Twitter account), it remains important to consider time and energy costs associated with these platforms, such that the set-up, monitoring, and maintenance of an individual or group’s presence on a platform can be time consuming. Future researchers should consider studying the return on investment for such efforts in knowledge translation and educational goals.

Looking ahead, researchers might also consider alternative framing for future studies. In many studies, we observed that researchers focused on a particular social media platform. However, some social media platforms (e.g. MySpace) rise and fall rapidly making these studies quickly obsolete. To extend the half-life of such studies, researchers should consider pushing the field towards more generalizable or transferrable concepts, such as identifying particular characteristics or features of platforms that align with educational theories or models.

## Conclusions

Our review found that social media platforms are ubiquitously used tools for knowledge translation and education targeting physicians and physician-trainees. Some best practices have emerged despite the transient nature of various social media platforms. Researchers and educators may find these media useful to engage with physicians and trainees to increase uptake of new knowledge and affect change in the clinical environment.

## Caption Electronic Supplementary Material

Appendix 1 – Scoping Review Extraction Tool
