# Supplementary material for: Social media in knowledge translation and education for physicians and trainees: a scoping review
Source: Perspect Med Educ. 2019 Dec 13;9(1):20–30. doi: 10.1007/s40037-019-00542-7 (PMC7012997; doi:10.1007/s40037-019-00542-7)
Supplement: Supplementary file 1 — Appendix 1 – Scoping Review Extraction Tool [file 40037_2019_542_MOESM1_ESM.pdf]

Appendix 1 - Scoping Review Extraction Tool

Scoping Review Extraction Tool

* Required

1. **Name of Extractor** *

2. **ID number of the paper** *

3. **Year of Publication** *

4. **Last name of first author:** *

5. **Does this paper meet our finalized inclusion criteria?** *
   Is this paper about knowledge dissemination, knowledge translation, or education of a medical professional (med student, resident, fellow, practicing physician)? RECALL - we are excluding papers purely about professionalism on social media. We are hoping to find out what are the social media (SoMe) based strategies employed by physicians to disseminate research (to themselves [aka KT or education or CPD], to learners [aka education], to patients, to the public?).
   Mark only one oval.

   - Yes
   - No *After the last question in this section, stop filling out this form.*
   - Maybe

6. **If you answered No or Maybe in 5a., what was your hesitation?**

Language Screen

7. **Is this paper in English?** *
   Mark only one oval.

   - Yes
   - No (The Principal Investigator will review these) *Stop filling out this form.*
   - No, but you are Principal Investigator doing the review

Data Extraction

8. **What type of scholarship was this?** *

For full description of these types, check out this paper: https://onlinelibrary.wiley.com/doi/abs/10.1111/j.1365-2923.2007.02974.x For paper that are possibly both, please use the OTHER option and type the two types in.
Mark only one oval.

- Conceptual piece (i.e. narrative review, opinion, editorial, or other theory advancing commentary) *After the last question in this section, skip to question 20.*
- Description of an innovation (What we did)
- DESCRIPTION STUDY (usually of a phenomenon): focuses on the first step in the scientific method, namely, observation. e.g. Intervention descriptions outcome data reported, usually no comparisons.
- JUSTIFICATION STUDY: focuses on the last step in the scientific method by comparing one educational intervention with anot her to address the question(often implied): "Does the new intervention work?"
- CLARIFICATION STUDY: employ each step in the scientific method, starting with observations (typically building on prior research) and model s or theories, making predictions, and testing these predictions.
- Other: ________________

9. **Was there a specific specialty featured? If so, what?**

e.g. Urology, Emergency Medicine

____

10. **What types of audiences were most discussed within this paper?** *

Check all that apply. e.g. If they are discussing adoption of new evidence in practicing physicians, click the Residents and Attending physicians
Check all that apply.

- [ ] Unknown
- [ ] Medical Students NOS - i.e. Level NOT specified
- [ ] Pre-clerkship Med Students
- [ ] Clerkship Med Students
- [ ] Residents/Fellows (Trainees who are practicing)
- [ ] Attending Physicians (Practicing qualified physicians)
- [ ] Basic or Non-Clinician Scientists
- [ ] Nurses
- [ ] Other healthprofessionals (OT, PT, Paramedics, Midwives - please clarify in "Other")
- [ ] Other: ________________

Data Extraction re: Studies or Innovation Reports

11. **Item of Interest:** *

Person, blog, podcast, video.
Mark only one oval.

- ( ) Person (Trainee, Clinician, etc..)
- ( ) Technology (Blog post etc..)
- ( ) Other: ____________________

12. **Population Size (Sample Size):**

Please describe the item of interest AND additional numbers etc. If you can write SMALL SCALE (Local study), MEDIUM SCALE (multi-centre), LARGE SCALE (MOOC)

____

13. **Timespan of DATA collection**

____

14. **In which continent(s) was study performed (select all that apply)?** *

Check all that apply.

- [ ] N/A (e.g. Internet-based, commentary)
- [ ] Africa
- [ ] Asia
- [ ] Middle East
- [ ] Australia/New Zealand
- [ ] Europe
- [ ] North America
- [ ] South America
- [ ] Other: ____________________

15. **Social Media that was emphasized** *

Check all that apply. If they list "#FOAMed" please discern which media they refer to (if they mention hashtags then likely twitter, but may be a combination of blogs. Must be a bulk of the feature of interest. MUST BE MENTIONED IN THE METHODS or for reviews mentioned more than 5 times.
Check all that apply.

- [ ] Twitter
- [ ] Facebook
- [ ] Google+
- [ ] Wiki
- [ ] WhatsApp
- [ ] WeChat
- [ ] Video Chat (Skype, Google Hangouts)
- [ ] Video Archival (e.g. YouTube, Vimeo)
- [ ] Photosharing (e.g. Flickr, Pixabay, Picasa)
- [ ] Snapchat
- [ ] Instagram
- [ ] Reddit
- [ ] Blogs
- [ ] Podcasts
- [ ] RSS feeds
- [ ] Collaborative Platforms (Google Docs, Dropbox)
- [ ] Other: ______________________

16. **What types of populations were the target of the study, case report, or intervention?** *

Check all that apply.
Check all that apply.

- [ ] Unknown
- [ ] Pre-clerkship Med Students
- [ ] Clerkship Med Students
- [ ] Residents/Fellows (Trainees who are practicing)
- [ ] Attending Physicians (Practicing qualified physicians)
- [ ] Basic or Non-Clinician Scientists
- [ ] Nurses
- [ ] Other healthprofessionals (OT, PT, Paramedics, Midwives - please clarify in "Other")
- [ ] Other: ______________________

17. **Theme** *

What was/were the major theme(s) of the article? (Check all that apply)
Check all that apply.

- [ ] Professionalism
- [ ] Practice improvement (e.g. improving knowledge, changing behaviour)
- [ ] Description of Technology (e.g. Describes how you might use blogs, wikis, etc)
- [ ] Evidence-based practice (e.g. journal club, critical appraisal of journal articles)
- [ ] Critical Appraisal of Online Resources (e.g. rating quality of websites, blogs, podcasts, twitter)
- [ ] Acceptability (Kirkpatrick Level 1)
- [ ] Demonstrating Learning (Kirkpatrick Level 2)
- [ ] Behaviour Change (Kirkpatrick Level 3)
- [ ] Organizational Performance (Kirkpatrick Level 4)
- [ ] "Community of Practice" or other synonyms (online community, etc..)
- [ ] Other: ______________________

18. **What data sources were used?** *

Click N/A if it doesn't apply.
Check all that apply.

- [ ] N/A
- [ ] Usage analytics (Web or Social Media Platform analytics - Pageviews, Tweets, etc..)
- [ ] Substantive written texts (narratives, reflections, blog posts)
- [ ] Micro-text analysis (e.g. Tweet analysis)
- [ ] Papers (literature review)
- [ ] Surveys (quantitative - number-based, questions Likert scales, other scales)
- [ ] Surveys (qualitative - open ended questions)
- [ ] Objective observations/Tests
- [ ] Interviews
- [ ] Focus Groups
- [ ] Downloads (PDFs, files)
- [ ] Ethnographic approaches (e.g. Observations with field notes, online observation)
- [ ] Simulation (e.g. Time to completion)
- [ ] Other: ______________________

19. **Methodology** *

For innovation reports, look at the results section of the program evaluation to determine what methods were used. If none were reported (i.e. innovation report without evaluation, then click the 4th option below to indicate it was a Descriptive Innovation Report only).

Mark only one oval.

- ( ) Quantitative *Skip to question 27.*
- ( ) Qualitaitve *Skip to question 25.*
- ( ) Mixed methods *Skip to question 25.*
- ( ) Descriptive innovation report ONLY with no actual outcomes data. *Skip to question 30.*
- ( ) Other: ______________________ *Skip to question 30.*

Data Extraction for Conceptual/Commentary Pieces

20. **Item/topic of Interest:** *

Person, blog, podcast, video.

Mark only one oval.

- ( ) Person (Trainee, Clinician, etc..)
- ( ) Technology (Blog post etc..)
- ( ) Other: ______________________

21. **Regarding which continent(s) was piece about (select all that apply)?** *

Check all that apply.

- [ ] N/A (e.g. Internet-based, commentary)
- [ ] Africa
- [ ] Asia
- [ ] Middle East
- [ ] Australia
- [ ] Europe
- [ ] North America
- [ ] South America
- [ ] Other: ______________________

22. **Social Media that was emphasized** *

Check all that apply. If they list "#FOAMed" please discern which media they refer to (if they mention hashtags then likely twitter, but may be a combination of blogs. Must be a bulk of the feature of interest. MUST BE MENTIONED IN THE METHODS or for reviews mentioned more than 5 times.

Check all that apply.

- [ ] Twitter
- [ ] Facebook
- [ ] Google+
- [ ] Wiki
- [ ] WhatsApp
- [ ] WeChat
- [ ] Video Chat (Skype, Google Hangouts)
- [ ] Video Archival (e.g. YouTube, Vimeo)
- [ ] Photosharing (e.g. Flickr, Pixabay, Picasa)
- [ ] Snapchat
- [ ] Instagram
- [ ] Reddit
- [ ] Blogs
- [ ] Podcasts
- [ ] RSS
- [ ] Collaborative Platforms (Google Docs, Dropbox)
- [ ] Other: ____________________

23. **Theme** *

What was/were the major theme(s) of the article? (Check all that apply)

Check all that apply.

- [ ] Professionalism
- [ ] Practice improvement (e.g. improving knowledge, changing behaviour)
- [ ] Description of Technology (e.g. Describes how you might use blogs, wikis, etc)
- [ ] Evidence-based practice
- [ ] Acceptability (Kirkpatrick Level 1)
- [ ] Demonstrating Learning (Kirkpatrick Level 2)
- [ ] Behaviour Change (Kirkpatrick Level 3)
- [ ] Organizational Performance (Kirkpatrick Level 4)
- [ ] "Community of Practice" or other synonyms (online community, etc..)
- [ ] Other: ____________________

24. **What data sources were used?** *
Click N/A if this does not apply.
Check all that apply.

- [ ] N/A
- [ ] Substantive written texts (narratives, reflections, blog posts)
- [ ] Micro-text analysis (e.g. Tweet analysis)
- [ ] Papers (literature review)
- [ ] Surveys (quantitative - number-based, questions Likert scales, other scales)
- [ ] Surveys (qualitative - open ended questions)
- [ ] Objective observations/Tests
- [ ] Interviews
- [ ] Focus Groups
- [ ] Downloads (PDFs, files)
- [ ] Ethnographic approaches (e.g. Observations with field notes, online observation)
- [ ] Other: ____________________

Skip to question 30.

Qualitative

25. **What was the specific qualitative study design?** *
Use what author says; add notes in "Other" if questions or disagree with authors
Check all that apply.

- [ ] Case Study
- [ ] Discourse analysis
- [ ] Ethnography (including online ethnography)
- [ ] Grounded theory
- [ ] Phenomenology
- [ ] Thematic analysis
- [ ] Program Evaluation - Survey free text
- [ ] Program Evaluation - interviews, focus groups
- [ ] Other: ____________________

26. **Did you review a mixed methods paper?** *
Mark only one oval.

- ( ) Yes *Skip to question 27.*
- ( ) No *Skip to question 30.*

Quantitative Method

27. **What was the specific quantitative study design? Select all that apply.** *
(Use what author says; add notes in “Other” if questions or disagree with authors).
Check all that apply.

- [ ] Descriptive
- [ ] Inferential (including psychometrics)
- [ ] Retrospective
- [ ] Prospective
- [ ] Program Evaluation
- [ ] Other: ______________________

28. **Was this study an EXPERIMENT?** *
i.e. in the methods do they specifically described as a RCT, or an experiment where they intervened at least one part of the population? (E.g. gave people access to a twitter account?)
Mark only one oval.

- ( ) Yes *Skip to question 29.*
- ( ) No *Skip to question 30.*
- ( ) Unsure *Skip to question 29.*

Skip to question 30.

Experimental Methods

29. **What was the specific experimental study design? Select all that apply. (Use what author says; add notes in “Other” if questions or disagree with authors).**
Check all that apply.

- [ ] Randomized controlled trial
- [ ] Pre-test / Post-test
- [ ] Single group, no comparison
- [ ] Single group, repeated measures
- [ ] Other: ______________________

Final questions

30. **Anything interesting or curious about this paper?**

____
____
____
____
____

31. **Were you unsure about this paper in any way?**

32. **Is this article a potential candidate for discussion as an exemplar?** *
Exemplar papers are outstanding examples that illustrate some key facet (E.g. it is a great descriptive paper).
Mark only one oval.

- Yes
- No
- Maybe

33. **If yes, explain why?**

34. **Should this article be EXCLUDED from the review?** *
Mark only one oval.

- No
- Maybe
- Yes

35. **If yes/maybe, explain why?**

Powered by
Google Forms
